# Management and Analysis of Sports Health Level of the Elderly Based on Deep Learning

**DOI:** 10.1155/2022/6044320

**Published:** 2022-06-30

**Authors:** Liping Xiao, Limin Huang, Hongxia Chang, Li Ji, Ji Li

**Affiliations:** ^1^College of Sports Science, Harbin Normal University, Harbin 150025, China; ^2^College of Sports and Human Sciences, Harbin Sport University, Harbin 150008, China; ^3^Administration of Sport for Persons with Disabilities, Beijing 101318, China

## Abstract

With the accelerating rate of population aging in China, the health of the elderly has received more and more attention and has become one of the most important issues in the elderly care industry. Because of insufficient research on the personal health of the elderly, the value of medical examination data cannot be fully exploited, many physical indicators have a certain impact on overall health or heart health, and there are few studies on heart health assessment. This paper proposes a deep learning-based elderly management analysis method of human exercise health level, using the exercise health management model to evaluate the heart health level of the elderly. Firstly, the indicators to measure heart health are proposed through traditional expert knowledge and personal health index to analyze heart health. Through dynamic assessment, predict the heart health status at the next time point, analyze possible heart diseases, and provide corresponding methods for the health of the elderly, which helps improve the physical health of the elderly. Quality of life provides assistance to meet the needs of improving the health of older adults.

## 1. Introduction

Studies have confirmed that long-term regular exercise in the mode of exercise health management can significantly improve the heart health of the elderly and improve the physical health and quality of life of the elderly [1–3]. In the late 1950s, the United States first proposed the concept of health management. Its core content is that medical insurance institutions carry out systematic health management for their medical insurance customers (including disease patients or high-risk groups), including disease prevention, All aspects of clinical diagnosis and treatment, rehabilitation, and health care are the whole process of comprehensive monitoring, analysis, evaluation, prediction, prevention, and maintenance of health risk factors of healthy people, subhealthy people, and diseased people and strive to effectively control the occurrence or development of diseases, significantly reducing the probability of an accident and actual medical expenses, thereby reducing the purpose of medical insurance compensation losses [4–9]. This is generalized health management. In a relatively narrow sense, health management (Heath Management) refers to the purpose of preventing and controlling the occurrence and development of diseases, reducing medical expenses, and improving the quality of life [10–15]. Chinese scholars have combined several representative definitions of health management at home and abroad to define health management as a process of comprehensively managing the health needs of individuals and groups [16–19]. Here, health needs can be a health risk factor, such as hypertension and obesity, or a disease state, such as coronary heart disease or diabetes. In short, it is the process of planning, organizing, directing, coordinating, and controlling health resources for health needs [2].

The United States is a pioneer in promoting health through exercise. Since 1980, exercise has been incorporated into the health management system. After years of development, the United States has established an exercise promotion and health guidance service platform that is led by the government, supplemented by scientific research institutions and sports social organizations, and combines medical and health services with physical fitness services [20–24]. Based on inspecting the American sports promotion health guidance service platform, from the establishment of the “medicine and body integration” linkage management mechanism, to play the role of social organizations such as the Chinese Society of Sports Science, to refine the implementation goals of sports to promote health, and to strengthen compound sports to promote health. My country has built a national fitness service system in terms of guiding personnel training and other aspects. Physical exercise is an important embodiment of a healthy lifestyle, but exercise must also have a degree, that is, scientific exercise management methods are needed. Sports health management is to apply modern medical knowledge and scientific sports methods to systematically pay attention to and maintain the physical health of sportsmen from the perspectives of sociology, physiology, and psychology. Sports health management refers to understanding the needs and goals of sports from physical fitness assessment, sports consultation, and communication based on the health status, living status, and exercise habits provided by the individual, establishing a personal exercise prescription, and providing safe, effective, Reasonable exercise plan, guided by practical teaching methods, combined with comprehensive health services for exercise effect tracking, help improve personal physical fitness and fitness, and then achieve the goal of personal exercise and fitness and physical health.

Existing studies have shown that sports health management is based on the change in people's health outlook. We review the research on sports health management, then expound on the basic elements of the public sports health management service model and the relationship between the elements, and demonstrate the operation mechanism of the service model and make corresponding recommendations. According to the health status, living status, and exercise habits provided by the individual, from physical fitness assessment, exercise consultation, and communication, to understand their exercise needs and goals, establish a personal exercise prescription, and provide a safe, effective, and reasonable exercise plan to Practical teaching methods are provided for guidance, combined with a full range of health services to track exercise results, to help improve personal physical fitness and fitness, and then achieve the goal of personal exercise and fitness and physical health. Some studies prove that exercise can enhance physical fitness, promote the overall development of the human body, enhance the human body's resistance and prolong life, and improve the health level of patients with certain diseases; it is a kind of active rest, which makes people energetic and relieves life, work, and study tension and improves work efficiency. Exercise can effectively prevent psychological disorders, enhance the joy of life, and improve the quality of life. Exercise is an important part of the health management process. In the development of a healthy experiment plan, the three aspects of healthy exercise, healthy diet, and health coaching can be combined. Physical activity monitoring is also a part of health monitoring, exercise health management is a part of exercise application in health management, and exercise prescription is a part of exercise health management. Health managers can guide and formulate healthy exercise for different groups of people according to the basic procedures of the survey.

As the elderly grow older, their physical condition also changes. The function of the heart will gradually deteriorate with aging, the systemic decline and functional degeneration of the elderly, and the immune system function of the elderly will decline significantly. The decline of the immune system function will directly affect the healthy life of the elderly, and the exercise of the elderly. The characteristics are also inseparable from their physiological characteristics; low-intensity, aerobic exercise for the elderly should not choose speed-type and strength-type sports, but whole-body sports that focus on aerobic metabolisms, such as Health Qigong, jogging, walking, and other projects.

To sum up, this paper proposes an analysis method for the management of the elderly's exercise health level based on deep learning. Based on traditional expert knowledge and personal health index, it proposes indicators to measure heart health to analyze heart health. Sequence data set, perform a dynamic assessment of heart health, obtain the possible heart health status at the next time point, remind the risk of heart disease, and make appropriate adjustments to the body in time. Human physical health provides corresponding methods to help improve the physical health and quality of life of the elderly, so as to meet the needs of improving the health of the elderly.

## 2. Dynamic Assessment of Heart Health in the Elderly

### 2.1. Heart Health Indicators for the Elderly

Heart health problems in older adults are complex problem. As age continues to increase, the coordination ability between the various tissues of the human body will also decrease. Heart disease is a high-risk disease that threatens the health of the elderly. Pathogenesis is complex, and many indicators affect the health of the heart. Numerous studies have shown that senile heart disease mainly includes three types of hypertensive heart disease, coronary heart disease, and pulmonary heart disease. During the development of these three types of heart disease, the contraction force of the heart muscle will be weakened, which will cause a circulatory disturbance, become heart failure, and be life-threatening. In addition, these heart diseases may also cause heart rate disorder, which is manifested as a fast, slow, or irregular heartbeat. Therefore, in the process of studying the heart health of the elderly, it is necessary to analyze from multiple angles and multiple indicators. Heart disease is not diagnosed casually. It is not an arrhythmia or angina pectoris during exercise. It means that you have heart disease. Its diagnosis requires a professional doctor to use the diagnostic criteria of heart disease to judge. There are many manifestations of heart disease, such as cardiovascular problems and myocardial infarction problems, that is, individuals may suffer from coronary heart disease [25–27].

Heart health indicators are based on a combination of traditional expert knowledge, factors that cause senile heart disease, and personal health indexes. Most of the research in the field of heart disease is to predict the risk of heart disease or use a single attribute feature to analyze heart health changes, but few scholars start from multiple indicators and comprehensively consider changes in heart health. The detailed index description is shown in Table 1.

From the perspective of professional medicine, indicators such as blood pressure, cholesterol, and blood sugar play a very important role in the diagnosis of heart disease, and heart health is analyzed from a more comprehensive perspective, and relevant indicators need to be considered more comprehensively. And the rapid development of big data technology makes it possible to obtain knowledge from more physiological indicators.

### 2.2. Heart Health Dynamic Assessment Questions

**Figure 1:**
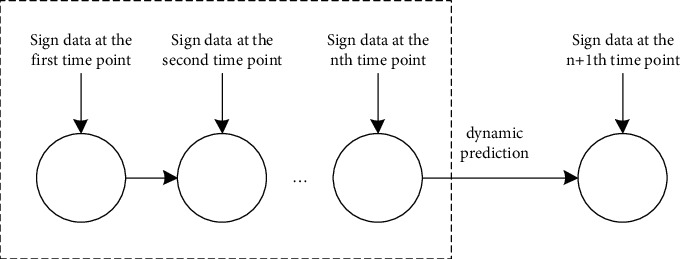
Detailed schematic diagram of dynamic assessment of heart health.

For the elderly, as the age continues to increase, the functions of various tissues in the body continue to decline, and the body's self-regulation ability will also decrease. Once a certain index is abnormal, it is likely to occur in a short period changes in health status. For older adults without heart disease, changing data on signs of heart health may lead to an increased risk of heart health and, in turn, heart disease. For older adults with pre-existing heart disease, changing data on signs related to heart health may lead to worsening heart disease. In this section, heart health is dynamically assessed according to the continuous changes in the physical data. The detailed schematic diagram is shown in Figure 1, which means that the physical data information of the previous *n* time points is used to predict the heart health status of the next time point. Dynamic evaluation, that is, to evaluate the possibility that the heart health state will evolve in a good or bad direction based on the sign data information at different time points.

Based on the above description of the problem of dynamic assessment of heart health, this section will analyze the dynamic assessment method and data preprocessing stage. Since it is a time-series dataset with multi-attribute features, the long short-term memory network (LSTM) in the field of deep learning has a good performance in processing long-term series data. But the input layer of the LSTM algorithm must be in canonical form, containing three dimensions, as follows.

Sample: all features at any time point are used as a single sample, and one or more samples constitute a batch.

Time step: each time point is a time step.

Features: the observed results for one-time step are called features.

**Definition 1 .**

*Series1*:*x*_11_,*x*_12_,*x*_13_,…,x_1n_,*Series2*:*x*_21_,*x*_22_,*x*_23_,…,x_2n_,*Seriesm*:*x*_m1_,*x*_m2_,*x*_m3_,…x_mn_, are attribute sets of *n* features at *m* time points.

Before entering the LSTM, the dataset needs to be transformed into a 3D collection using the reshape method. There are a total of *n* time points, so the step size is *n*. Then the LSTM input layer input_shape (1, n, m). For the heart health data set, the vector similarity method is used to simulate a data set containing 5 time points, a total of 6166 individual data, so the input layer input_shape (5, 14, 5). The heart health state at the next time point is predicted from the personal sign data of the first *n* time points. The training set constructed is as follows:

(1) $$\begin{array}{c} \text{train}\_X=\left[\begin{array}{ccc} x_{11} & \ldots & x_{1\left(n-1\right)}\\ \ldots & \ldots & \ldots \\ x_{\left(m-1\right)1} & \ldots & x_{\left(m-1\right)\left(n-1\right)} \end{array}\right],\\ {\text{train}}_{Y}=\left[\begin{array}{c} x_{1n}\\ \ldots \\ x_{mn} \end{array}\right]. \end{array}$$

The test set is constructed in the same way as the training set.

## 3. Deep Learning Algorithms

### 3.1. RNN Neural Network

Often used in machine vision and natural language processing, recurrent neural networks (RNNs) have feature learning capabilities and are able to extract high-level features from input data. One-dimensional CNN (1D-RNN) can be used for time-series data processing, and two-dimensional CNN (2D-RNN) can be used for visual processing such as image recognition.

**Figure 2:**
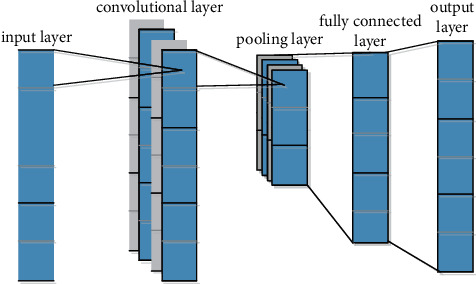
Structure of 1D-RNN.

The structure of 1D-RNN is shown in Figure 2, which consists of an input layer, a convolutional layer, a pooling layer, a fully connected layer, and an output layer. The convolutional layer extracts features through convolution kernels of different sizes, and the pooling layer reduces the dimension of information by compressing the data. To efficiently extract and retain data features, convolutional layers, and pooling layers alternate. Fully connected layers will flatten the distribution features extracted from different spaces to achieve regression or classification. RNN focuses on local feature extraction and reduces weights through parameter sharing, which greatly reduces the computational parameters of the network.

### 3.2. LSTM Neural Network

**Figure 3:**
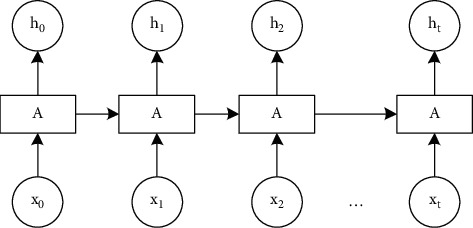
LSTM network structure diagram.

Since there are many time points in the time-series data of the dynamic assessment of mental health of the elderly studied in this paper, it belongs to the long-term dependence problem. If RNN is used, there may be a problem that the gradient disappears during the backpropagation process. The gradient is the value used to update the weights during the neural network training process. If the gradient disappears, the training process will not continue. Therefore, this paper uses LSTM for research. The basic LSTM network structure is shown in Figure 3.

**Figure 4:**
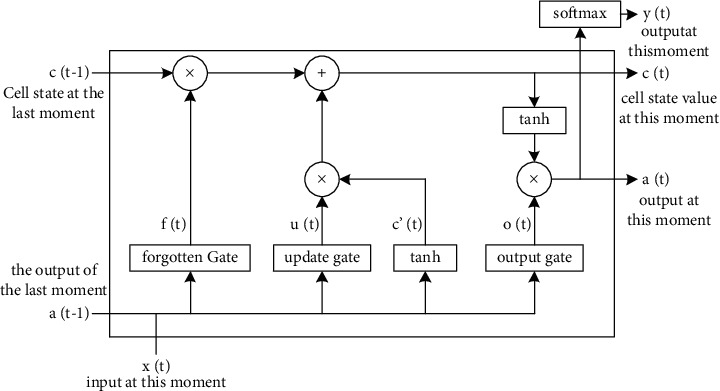
Detailed structure diagram of the LSTM computing unit.

As can be seen from Figure 3, *x*_t_ represents the input of the data of the input layer, *h*_t_ represents the output of the data of the output layer, A represents the operation of each node in the hidden layer, and there is certain information transmission between the operations of different nodes. The structure is shown in Figure 4.

As shown in Figure 4, combined with Figure 3, a complete LSTM network structure diagram includes the input layer, forget gate, update gate and output gate. During the learning process, the LSTM network structure will automatically store the historical information deemed useful to the *c*(*t*), which represents the memory of the current LSTM unit, *c* (*t*−1) represents the memory of the previous LSTM unit, *x* (*t*) represents the input at time *t*, where “+” represents the added information, and “×” represents the scaling Information. The activation functions of the forget gate, update gate, and output gate are all sigmoid because the information processed by the sigmoid function will become 0 or 1, which can decide which information is forgotten or remembered after passing through the node. In addition, since the second derivative of tanh has a long distance when it keeps approaching zero, it can better overcome the problem of gradient disappearance during training. The formula for calculating the update gate at time *t*:

(2) $$\begin{array}{c} u\left(t\right)=\text{Sigmoid}\left(W_{i}\left[x\left(t\right);a\left(t-1\right)+b_{i}\right]\right), \end{array}$$

where *W*_*i*_ is the weight of the update gate and *b*_i_ is the bias vector of the update gate.

The forgetting ratio in the forget gate is calculated as shown in

(3) $$\begin{array}{c} f\left(t\right)=\text{Sigmoid}\left(W_{t}\left[x\left(t\right);a\left(t-1\right)\right]\right)+b_{t}, \end{array}$$

where *W*_t_ is the weight of the forget gate and *b*_t_ is the bias vector of the forget gate. And the unit state at this moment is updated, as shown in

(4) $$\begin{array}{c} c\left(t\right)=f\left(t\right){}^{*}c\left(t-1\right)+u\left(t\right){}^{*}\;\tanh\left(W_{s}\left[x\left(t\right);a\left(t-1\right)\right]+b_{s}\right), \end{array}$$

where *W*_s_ and *b*_s_ are the weight and bias vector of hidden units in the forget gate,respectively.

Similarly, the update of the output gate information can be obtained, as shown in

(5) $$\begin{array}{c} o\left(t\right)=\text{Sigmoid}\left(W_{o}\left[x\left(t\right);a\left(t-1\right)\right]\right)+b_{o}, \end{array}$$

(6) $$\begin{array}{c} y\left(t\right)=o\left(t\right){}^{*}\;\tanh\left(c\left(t\right)\right). \end{array}$$

### 3.3. Deep Neural Network (DNN)

**Figure 5:**
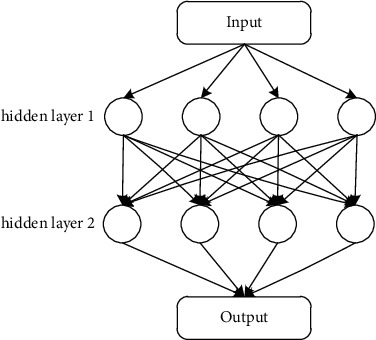
Schematic diagram of the 2-layer DNN network structure.

In simple terms, DNN is an artificial neural network with multiple hidden layers, and the layers are fully connected. The model training process consists of two parts, one part is the forward propagation process, that is, starting from the input layer, calculating layer by layer until the calculation reaches the output layer. The other part is the backpropagation process. Before backpropagation, the loss between the predicted value and the output value needs to be calculated first, and then the loss function is optimized to find the minimum extreme value. The most common use of the gradient descent algorithm, and then reverse Update a series of W weight matrices and B bias vectors until the desired effect is achieved and stop training. The structure diagram of the 2-layer DNN network is shown in Figure 5.

### 3.4. LSTM-DNN Model Construction

**Figure 6:**
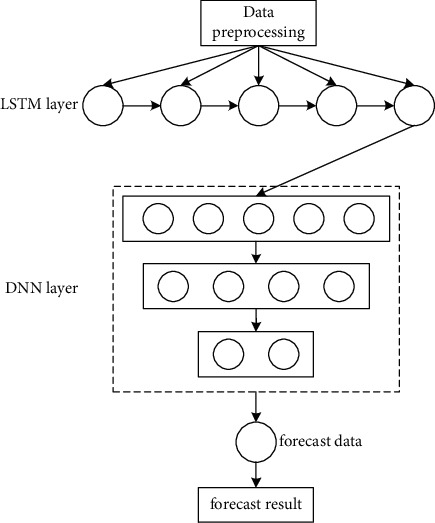
LSTM-DNN model structure diagram.

This paper will build the LSTM-DNN model and analyze it mainly from two aspects. First, the long-term sequence data set consider using the LSTM algorithm to learn the relationship between nodes at different times and make predictions; second, consider the particularity of the sign data because there may also be a certain relationship between different indicators, so use the DNN algorithm to learn the relationship between different indicators. The LSTM-DNN model structure is shown in Figure 6.

**Figure 7:**
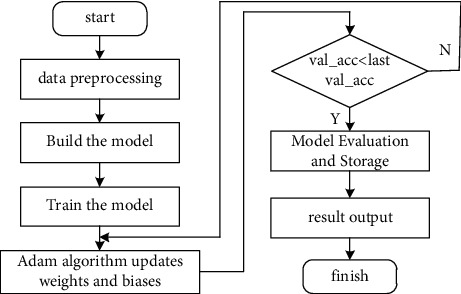
Model running flow chart.

First perform data preprocessing, then input to the LSTM layer, then enter the DNN layer, and finally output a prediction result. The flow chart of the model operation is shown in Figure 7.

**Step 1 .**

Data preprocessing. Using the maximum and minimum normalization principle, the original data is normalized to the range of [0, 1] according to the index, and the normalization formula is given by

(7) $$\begin{array}{c} x{}^{\prime }=\frac{x-x_{\min}}{x_{\max}-x_{\min}}, \end{array}$$

where *x*_max_ is the maximum value of the indicator, *x*_min_ is the minimum value of the indicator, and *x* is the value of the indicator for different sample data.

**Step 2 .**

Build a network training model. Assuming that the number of time points in the time dimension is *n*, the number of nodes in the LSTM layer is *n*. To solve the problem of gradient disappearance during the training process and improve the training speed of the model, the activation function uses ReLU. In the DNN layer, its essence is a fully connected layer, the number of hidden layers is set according to the size of the data, the activation function used is Sigmoid, and finally, the nodes of the output layer are set according to the category, if there are *n* categories, that is, set *n* node. After configuring the network structure, a loss function needs to be defined to calculate the gradient and optimize it. The loss function uses the root mean square error method (RMSE), as shown in

(8) $$\begin{array}{c} f\left(x\right)=\max\left(0,x\right),\\ \text{RMSE}=\sqrt{\frac{1}{M}\sum_{i=1}^{M}{\left(y^{\left(i\right)}-y^{\prime \left(i\right)}\right)}^{2}}. \end{array}$$

Here, *y*^(*i*)^ is the original value of the sample data, y^'(i)^ is the predicted value of the model, *M* represents the size of the sample data, and *i* represents the ith sample.

**Step 3 .**

Train the network model. After completing the second step of network construction, start training, and configure parameters according to the size of the data set and empirical knowledge, including the number of iterations epoch, and the batch size of the data sent into the network each time, which is used for verification after each epoch. In the evaluation function of the model, the parameter is set to “accuracy”, is simply by judging the ratio of the number of correct predictions to the total number in the test set. The optimizer in the training process uses the Adam algorithm. When the accuracy rate on the test set continues to increase and tends to be stable, it reaches the standard of the end of model training, that is, stops the training.

**Step 4 .**

Model evaluation and saving. After the set parameters are verified, when the accuracy rate of the test set reaches a stable level, the model training is regarded as the end, and the accuracy rate at this time is the optimal accuracy rate. The final values of the parameters and the model that are continuously adjusted during the training process are saved for application to other samples.

**Step 5 .**

Predict the resulting output. During the training process, the result accuracy rate and loss value of each iteration are printed, which is convenient for debugging parameters, and finally, the root mean square error of the test set and the result accuracy rate are output.

## 4. Experiment and Analysis

The medical examination data set used in this study comes from the public data of the National Health and Nutrition Examination Center in the United States, and only the elderly part is used, with a total of 6166 pieces of data. When analyzing heart health, indicators based on expert knowledge are the indicators used to measure heart disease. The reference data set is the standard data set of heart disease published by UCI. In the model training process, the heart health is measured by whether there is a heart disease, and the disease is mapped to 0, that is, the heart health is poor; otherwise, it is mapped to 1. The dynamic evaluation process uses all the index information of the first four time nodes to dynamically predict the heart health status at the fifth time point. The experimental environment is windows10, python3.6, and the model is built using the Keras framework in deep learning.

**Figure 8:**
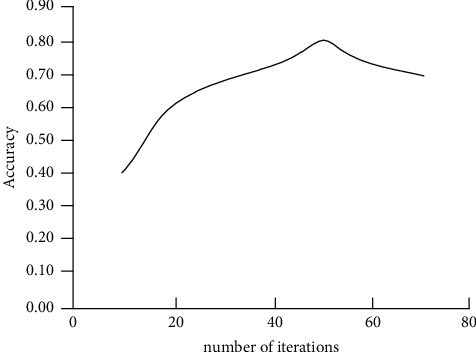
The number of iterations and their corresponding accuracy results.

Since the number of time nodes in the dataset is 5, the LSTM part is configured with 5 nodes to learn the relationship between different time nodes. After many experiments, the number of DNN hidden layers is 2, the number of nodes is 4 and 2 respectively, the node of the output layer is set to 1, and finally, a prediction result is obtained. According to the size of the data set and related experience, after many attempts, the Epoch is set to 60, and the number of iterations and the corresponding accuracy result. When the number of iterations reaches 55, the accuracy of the result is the best, which is 79.6%, as shown in Figure 8.

Use the model in this chapter to compare and analyze the RNN algorithm and the BPNN algorithm. The precision rate, recall rate, and F1 value are used as evaluation indicators for analysis and comparison, and the experimental results are shown in Table 2.

The experimental results show that the proposed LDNN model can better dynamically assess the state of heart health. The main reason is that the LSTM algorithm has better performance on long-term dependency problems and can better learn the correlation between different time points. And the result is processed by the DNN network structure with two hidden layers, and the relationship between the sample features is better learned, so the LDNN model constructed in this chapter has a better training effect and a higher prediction accuracy. Finally, in terms of the accuracy of the results, the LDNN model is 3% higher than the RNN algorithm on average and 7% higher than the BPNN algorithm.

## 5. Conclusion

Aiming at the heart health problems of the elderly in the elderly care industry, the related algorithms of deep learning are used to analyze and evaluate the heart health of the elderly. According to the time-series data set composed of indicators related to heart health, the problem of dynamic assessment of the heart health of the elderly is described and analyzed, and finally, the LSTM-DNN model is built for the dynamic assessment of the heart health of the elderly and combined with the RNN algorithm and BPNN The algorithm compares the experimental results, and the accuracy of the evaluation results is increased by 3% and 7% respectively, which verifies the effectiveness of the model. Through the method in this paper, the possible heart health status at the next time point can be obtained, and the risk of heart disease can be reminded, so as to make appropriate adjustments to the body in time and improve the quality of life of the elderly. The evaluation method research in this paper needs to be further improved: First, the data set comes from the data of Americans, which may be different from the physical data of Chinese people. Second, it is about the proposal of measuring heart health indicators. This paper only considers numerical data and does not involve text, type data, and image type data.

## Figures and Tables

**Table 1:** Detailed information table of indicators to measure heart health.

Metrics	Reference range
Age	60+
Gender	{Male, female}
Blood pressure level	Systolic blood pressure: 90–14 mmHg
	Diastolic blood pressure: 60–90 mmHg
Types of chest pain	{ angina pectoris, non-angina pectoris, classic angina pain, and atypical angina }
Serum cholesterol	2.9∼6.0 mmol/L
Blood sugar concentration	3.89∼6.1 mmol/L
ECG results	{ normal, rising, falling }
Resting heart rate	60∼100 times/min
Number of blood vessels	{0, 1, 2, 3}
PHI	[0, 1]

**Table 2:** Comparison of experimental results.

Algorithm	Correct rate	Recall	F1 value
BPNN	0.725	0.668	0.702
RNN	0.764	0.725	0.742
LSTM-DNN	0.796	0.776	0.785

## Data Availability

The dataset can be accessed upon request to the corresponding author.

## References

[1] Zhao W. (2019). Correlative analysis of occupational health management in construction companies to related factors based on fuzzy clustering and grey theory. *IOP Conference Series: Earth and Environmental Science*.

[2] Theilig M. M., Korbel J. J., Mayer G., Hoffmann C., Zarnekow R. (2019). SPECIAL SECTION ON DATA-ENABLED INTELLIGENCE FOR DIGITAL HEALTH—employing environmental data and machine learning to improve mobile health receptivity. *IEEE Access*.

[3] Zhang L., Lin J., Liu B., Zhang Z., Yan X., Wei M. (2019). A review on deep learning applications in prognostics and health management. *IEEE Access*.

[4] Bleich M. R. (2018). The professional development educator role in leading population health management. *The Journal of Continuing Education in Nursing*.

[5] Pandey A., LaMonte M., Klein L. (2017). Relationship between physical activity, body mass index, and risk of heart failure. *Journal of the American College of Cardiology*.

[6] Moon D. J., Lee S. I., Lee C. S., Kim G. C., Kang H. J., Yang Y. J. (2008). A Suggestion on evaluating personal health state: health index. *Journal of Biomedical Engineering Research*.

[7] Ling C., Xue L., Wang S. Mining personal health index from annual geriatric medical examinations.

[8] Chen L., Li X., Yang Y., Kurniawati H. (2016). Personal health indexing based on medical examinations: a data mining approach. *Decision Support Systems*.

[9] Jung H., Chung K. (2016). PHR based life health index mobile service using decision support model. *Wireless Personal Communications*.

[10] Esposito M., Minutolo A., Megna R., Forastiere M. M. G. (2018). A smart mobile, self-configuring, context-aware architecture for personal health monitoring. *Engineering Applications of Artificial Intelligence*.

[11] Swapna G., Kp S., Vinayakumar R. (2018). Automated detection of diabetes using CNN and CNN-LSTM network and heart rate signals. *Procedia Computer Science*.

[12] Maragatham G., Devi S. (2019). Retracted article: LSTM model for prediction of heart failure in big data. *Journal of Medical Systems*.

[13] Saadatnejad S., Oveisi M., Hashemi M. (2018). LSTM-based ECG classification for continuous monitoring on personal wearable devices. *IEEE Journal of Biomedical & Health Informatics*.

[14] Liu F., Zhou X., Cao J., Wang Z., Wang H., Zhang Y. A LSTM and CNN based assemble neural network framework for arrhythmias classification.

[15] Mohammed S., Sha Y., Wang M. D. “Early prediction of sepsis in EMR records using traditional ML techniques and deep learning LSTM networks.

[16] Brunese L., Martinelli F., Mercaldo F., Santone A. (2020). Deep learning for heart disease detection through cardiac sounds. *Procedia Computer Science*.

[17] López-Martínez F., Núñez-Valdez E. R., García-Díaz V., Bursac Z. (2020). A case study for a big data and machine learning platform to improve medical decision support in population health management. *Algorithms*.

[18] Hunter D. J., Brown J. (2007). A review of health management research. *European Journal of Public Health*.

[19] Yang Y., Tian C.-Huan, Cao J., Huang X.-J. (2019). Research on the application of health management model based on the perspective of mobile health. *Medicine*.

[20] Ahmad F., Guyeux C., Makhoul A., Ali J., Tawil R. (2018). On the coverage effects in wireless sensor networks based prognostic and health management. *International Journal of Sensor Networks*.

[21] Goe P., Liu H., Brown D., Datta A. (2008). On the use of spiking neural network for EEG classification. *International Journal of Knowledge-Based and Intelligent Engineering Systems*.

[22] Marino D. J. (2017). Building a value model for population health management. *Healthcare Financial Management : Journal of the Healthcare Financial Management Association*.

[23] Wei K., Zhou Z. (2020). Adversarial attentive multi-modal embedding learning for image-text matching. *IEEE Access*.

[24] Jia Z., Lin Y., Wang J., Ning X. (2021). Multi-view spatial-temporal graph convolutional networks with domain generalization for sleep stage classification. *IEEE Transactions on Neural Systems and Rehabilitation Engineering*.

[25] Lee Y. J., Ha (2016). Consumer use of the internet for health management. *Journal of Consumer Health on the Internet*.

[26] Zhen T., Yan, Yuan P. (2019). Walking gait phase detection based on acceleration signals using LSTM-DNN algorithm. *Algorithms*.

[27] Ospina J., Newaz A., Faruque M. O. (2019). Forecasting of PV plant output using hybrid wavelet‐based LSTM‐DNN structure model. *IET Renewable Power Generation*.

